# Cadaveric Study to Define the Anatomy of the Medial Patellofemoral Ligament (MPFL) and Its Variant Patterns

**DOI:** 10.7759/cureus.39333

**Published:** 2023-05-22

**Authors:** Sumit Patil, Mohtashim Ahmad, Manmohan Patel, Bertha Rathinam, Kawal K Pandita, John A Santoshi, Prateek Behera, Swapna B Parate

**Affiliations:** 1 Anatomy, All India Institute of Medical Sciences, Bhopal, Bhopal, IND; 2 Hospital Administration, All India Institute of Medical Sciences, Bhopal, Bhopal, IND; 3 Orthopaedics, All India Institute of Medical Sciences, Bhopal, Bhopal, IND; 4 Anatomy, SMBT IMS & RC, Igatpuri, Nashik, IND

**Keywords:** knee joint, ligament tear, knee ligament, patellar dislocation, medial patellofemoral ligament

## Abstract

Background: The medial patellofemoral ligament (MPFL) is one of the major soft tissue stabilizers on the medial side of the knee joint, extending from the medial condyle of the femur to the medial aspect of the patella. Different kinds of literature described different sizes and different origins and insertions of MPFL. Injury of MPFL causes patellar instability and dislocation. We reported the anatomy and morphology of MPFL and its implications in the repair of MPFL. The aim of the study was also to look at the variant forms of the MPFL.

Methodology: A total of 40 lower limbs fixed in formalin were dissected to study the MPFL of the knee. After reflecting the deep fascia and retinaculum on the medial side of the knee joint the MPFL was exposed. For better learning the lower medial part of vastus medialis was reflected, so that the part of MPFL undercover was exposed.

Results: Different forms of MPFL were seen like two straps 12.5%, broad rectangle 20%, and triangular shaped 67.5% MPFL. The origin of MPFL was found between the adductor tubercle and medial epicondyle of the femur and insertion was seen extending from the proximal medial half of the patella to the tendinous aponeurosis of vastus medialis obliquus (VMO) and vastus intermedius muscle (VIM).

Conclusions: This is the first study that described three variant patterns of MPFL in accordance with their morphological appearance. This knowledge will be helpful to the surgeons for easy identification and repair of the MPFL.

## Introduction

The patella is the largest sesamoid bone, embedded in the tendon of the quadriceps femoris. An expansion from the tendon of the quadriceps femoris blends distally with superficial fibers of the patellar ligament. From the superficial surface of the patella, the patellar retinaculum extends on the medial and lateral sides. A thickened band extending from the medial margin of the patella to the medial condyle of the femur and medial condyle of the tibia is called as medial patellofemoral ligament (MPFL) and medial patello-tibial ligament respectively [1]. These ligaments are important for the stabilization of the patellofemoral joint.

The MPFL is the major medial soft tissue restraint preventing lateral displacement of the distal knee extensor mechanism, contributing an average of 53% of the total force [2]. The MPFL is located within the second layer of the medial soft tissue of the knee, and it has a role in the prevention of lateral excursion of the patella [3]. Its femoral origin lies between the adductor tubercle and the medial epicondyle [4]. The MPFL widens from its narrow origin to the insertion covering approximately half of the proximal medial patella [5]. Numerous reconstructive techniques have been described using a variety of grafts, including hamstring tendons (gracilis or semitendinosus), portions of the quadriceps, or patellar tendons [6-8].

The aim and objectives of the study were to define accurately the anatomical aspect of MPFL and to bring up the different variant patterns of MPFL. The knowledge of MPFL anatomy and its different patterns will be helpful to the surgeons while performing the repair of the MPFL.

## Materials and methods

The study was conducted in the Department of Anatomy, AIIMS Bhopal, India. Forty limbs of cadavers aged between 48 and 90 years, fixed in formalin were used for the study. Permission through letter number IHEC-LOP/2021/IM0373 from the Institutional Ethical Committee was taken before starting the project. The specimens with pathological deformity, injury, or history of knee surgery were excluded from the study. The skin and superficial fascia on the anterior aspect of the distal part of the thigh, knee, and proximal part of the leg was reflected. The deep fascia of the front of the knee and medial aspect of the thigh was carefully removed to expose the muscles of the front of the thigh and retinaculum of the knee joint. The first layer of the retinaculum of the knee on the medial side was also removed. We traced the distal part of the medial border of vastus medialis muscle. It was carefully retracted to the lateral side to find the shiny fibrous structure extending from the medial condyle of the femur to the upper part of the patella. The adductor magnus tendon was also traced to its distal attachment at the adductor tubercle. The medial attachment of MPFL can be found in between the adductor tubercle and the medial epicondyle of the femur. Once the MPFL was identified, its lateral extent was defined by reflecting or cutting the distal part of vastus medialis muscle. The shape and pattern of fibers in MPFL were noted. The well-dissected specimens showing the variant pattern of MPFL were saved by capturing digital photographs. All 40 limbs after careful dissection showed the presence of MPFL. 

## Results

Anatomy of MPFL

Origin

**Figure 1:**
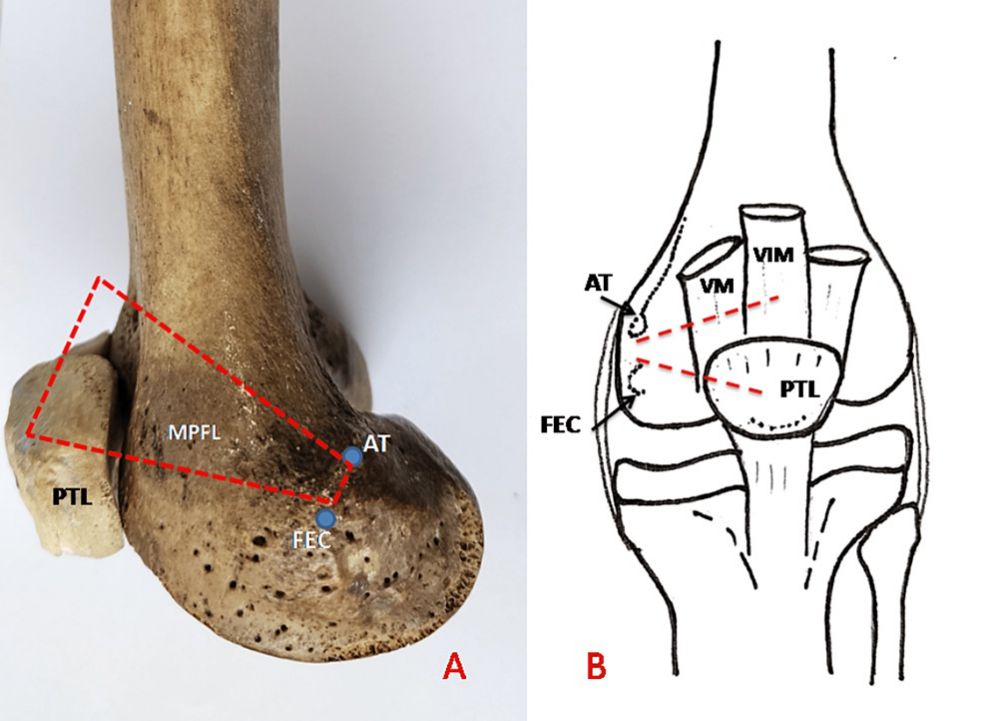
(A&B) Origin of MPFL from the area in between AT and FEC of the medial condyle of femur and insertion on proximal half of PTL and tendinous aponeurosis of VM and VIM muscles. Approximate position of MPFL is shown by red dotted lines. MPFL, medial patellofemoral ligament; AT, adductor tubercle; FEC, femoral epicondyle; PTL, Patella; VM, vastus medialis; VIM, vastus intermedius

Medial epicondyle of the femur (MEF) is a bony prominence found on the medial condyle of the femur and the adductor tubercle (AT) is a bony prominence present on the distal end of a medial supracondylar ridge of the femur. AT is superior and slightly posterior to the MEF. Origin of MPFL was found 10 ± 3 mm of breadth arising in between AT and medial epicondyle MEF as shown in Figure 1. AT was located closer to the MPFL as compared to MEF. The upper fibers of MPFL were arising very close to the lower part of the AT while the lower fibers of MPFL were 3-7 mm apart from the MEF depending on the breadth of the MPFL. 

Insertion

**Figure 2:**
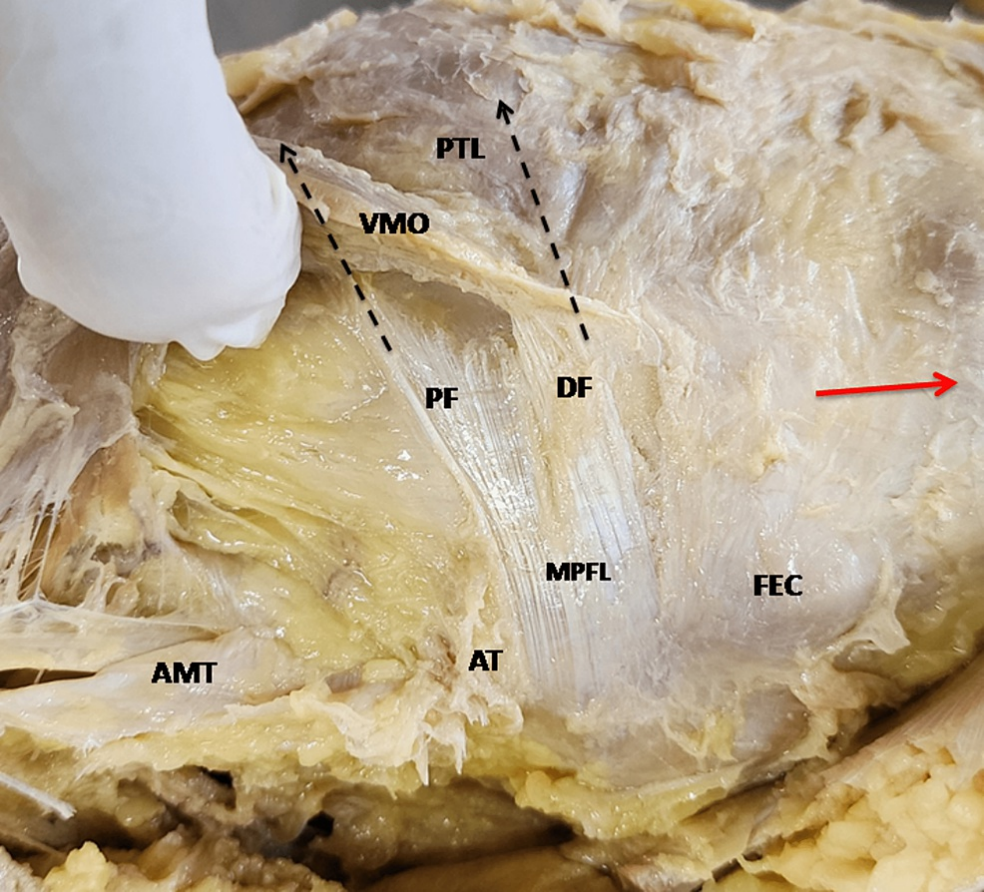
Direction of proximal and DF of MPFL. PF were adherent to the aponeurosis VMO, so the fibers broken while separating from VMO. DF, distal fibers; PTL, Patella; AMT, adductor magnus tendon; AT, adductor tubercle; FEC, femoral epicondyle; MPFL, medial patellofemoral ligament; PF, proximal fibers; VMO, vastus medialis obliquus The red arrow is directed distally towards the foot (medial side of the left knee).

Typically the fibers were seen directed medially upwards towards the superior medial half part of the patella. The MPFL was found deeper to vastus medialis obliquus (VMO). The fibers of MPFL were seen merging in the aponeurosis of VMO and vastus intermedius muscle (VIM). The insertion of MPFL has two parts, the proximal one merging in the tendinous aponeurosis of VMO and VIM and the distal part on the proximal medial half of the patella as shown in Figure 1. In the majority of cases, the proximal part was not tightly fixed with the aponeurosis of VMO and was separated by applying moderate force. In a few cases, it was tightly adherent, so it was broken while trying to separate it, as shown in Figure 2. 

Variant patterns of MPFL

We found three types of MPFL: typical triangular, rectangular, and two straps variety as shown in Table 1. 

**Table 1:** Variant patterns of the MPFL. MPFL, medial patellofemoral ligament

S. No.	Type of shape	Number of cases (40)	Number of cases in %
1	Triangular	27	67.5
2	Rectangular	8	20
3	Two straps	5	12.5

**Figure 3:**
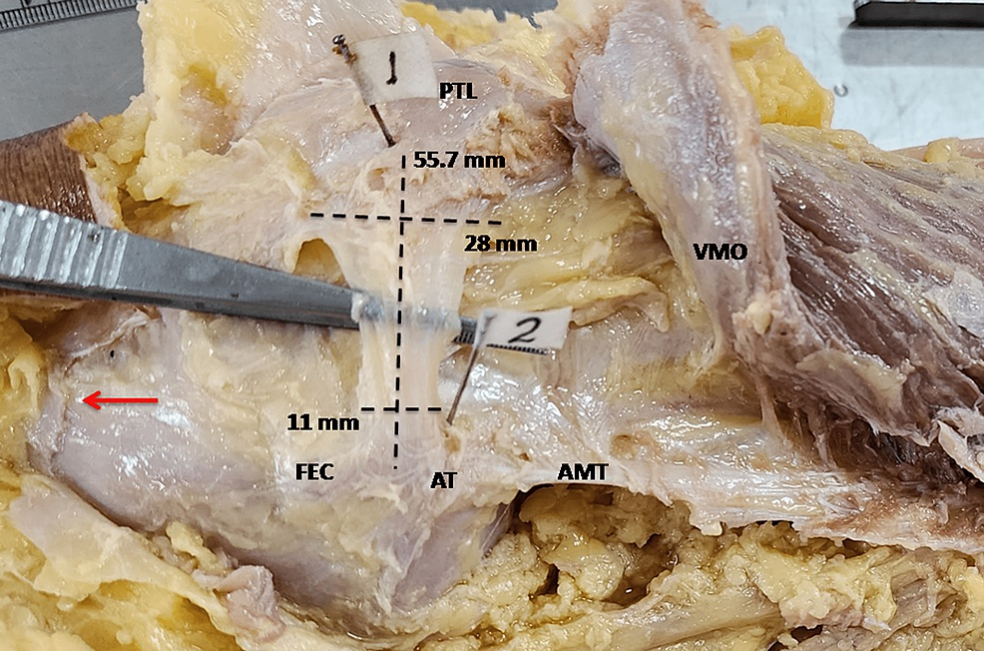
Typical triangular MPFL which had a length of 55.7 mm, width at origin lying between FEC and AT was 11 mm and width at PTL end 28 mm. For better exposure VMO was reflected. The red arrow is directed distally towards foot ( medial side of the right knee). MPFL, medial patellofemoral ligament; FEC, femoral epicondyle; AT, adductor tubercle; PTL, Patella; VMO, vastus medialis obliquus

**Figure 4:**
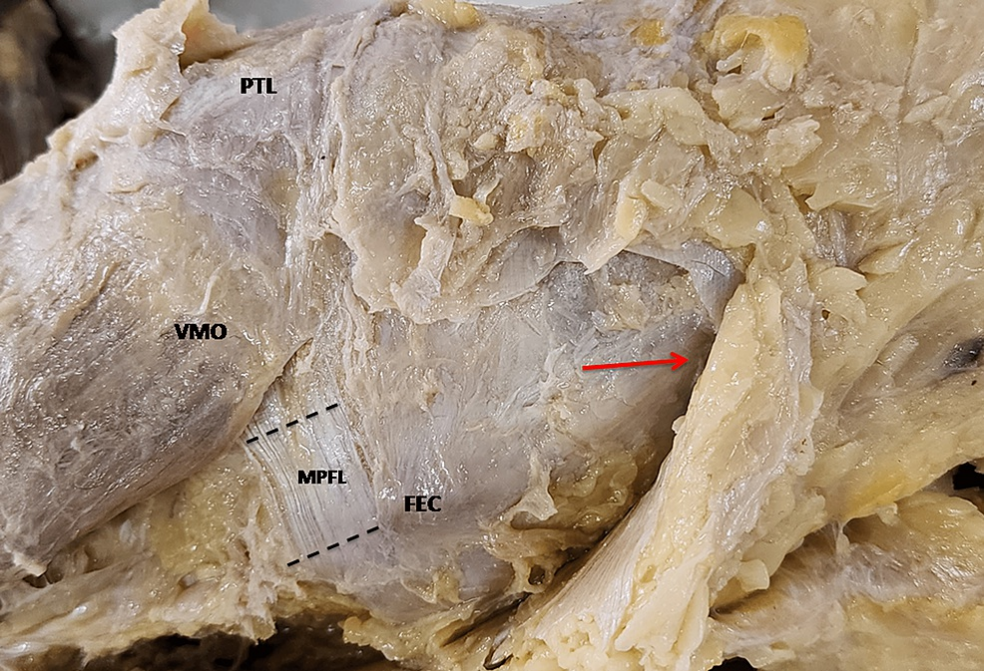
Rectangular variety of MPFL where width at medial and lateral end was roughly same and upper and lower borders seem to be parallel. MPFL, medial patellofemoral ligament; PTL, Patella; VMO, vastus medialis obliquus; FEC, femoral epicondyle. The red arrow is directed distally towards foot (medial side of left knee).

**Figure 5:**
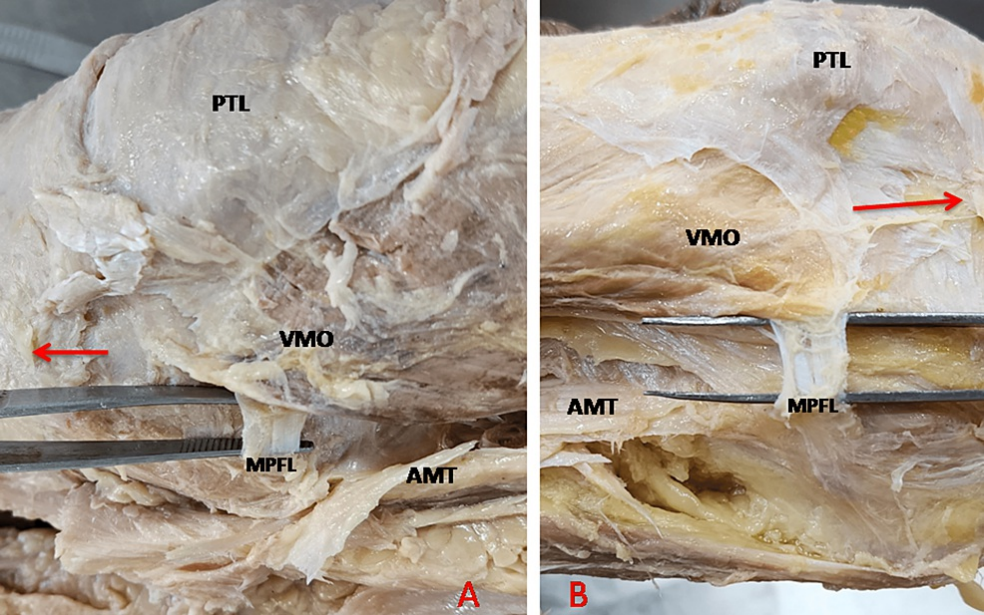
Two straps variety of MPFL. A: medial side of the right knee; B: medial side of the left knee MPFL, medial patellofemoral ligament; AMT, adductor magnus tendon; PTL, Patella; VMO, vastus medialis obliquus A red arrow is directed distally toward the foot

1. Typical triangular: This is typical shape of MPFL where the apex is located on the medial condyle of the femur and fanning fibres insert on a broad base represented by the upper medial half of the patella and quadriceps tendon as shown in Figure 3. At the femoral end, the mean width of MPFL is 11 mm and at the patellar end, it is 28 mm. The average length was 55.7 mm. We noticed 67.5% of MPFL of this variety.

2. Rectangular: In this variety, the broad sheet of fibers having an average width of 18 mm was seen extending from medial condyle of the femur to the medial border of the patella as shown in Figure 4. The upper and lower borders were roughly parallel to each other. This rectangular variant type of MPFL was found in 20% of knees. 

3. Two straps: The MPFL was in the form of two straps as shown in Figure 5. The average width of the two-striped MPFL was 15 mm in the middle. Slightly broadening was observed at the patellar end. This variant pattern was seen in 12.5% of limbs. 

## Discussion

Patellofemoral dislocations are common and occur due to quadriceps contraction across a flexed, valgus knee with the externally rotated weight-bearing tibia compared to the femur [9]. Conservative management focuses on concentric exercises to strengthen the quadriceps muscles and especially the vastus medialis to prevent further instability. Even after the attempts of rehabilitation, if the dislocation recurs; then operative intervention is needed with the aim of restoring the soft tissue anatomy of the medial side of the knee to normal. In all, 94% of patients suffer a tear to the MPFL following a patellar dislocation particularly on the lateral side [10].

In this study, we defined the different shapes of the MPFL which was traditionally considered triangular only. An attempt was also done to explain the exact location of origin and insertion of MPFL. Weber brothers were the first to describe the anatomy of the knee in 1836 and had no consensus on the anatomy of MPFL or its existence in the knee joint [11]. Nowadays there are multiple studies including this study, where 100% of knees showed the presence of MPFL [12-13]. 

Different studies described the different origins of MPFL, some from adductor tubercle [2-3, 14], and some from MEF [15]. Some authors reported the origin of MPFL was between the adductor tubercle and MEF and the same findings were noticed by us [4, 12]. Patellar insertion was found on the proximal half of the medial border [2, 5], and the proximal two third of the medial border [16-17]. Tuxøe et al. [17] reported that the MPFL was attached to the deeper part of the aponeurosis of VMO and the proximal two-third border of the patella. Baldwin [18] in his study found that the conjoint attachment of MPFL, first layer of retinaculum, and VMO tendon occupies the medial aspect of the patella corresponding to the articular surface of the patella which is present on the posterior aspect. They also concluded that the AT provided exclusively attachment to the adductor magnus tendon and MEF provided attachment to the medial collateral ligament. The origin of MPFL was from the groove in between AT and MEF. Mochizuki et al. [19] reported that the proximal fibers of MPFL were attached to the tendon of VIM without tight adhesions to VMO and distal fibers attached to the medial border of the patella. We also found the same results but in a few cases, there were tight adhesions between the proximal fibers of MPFL and VMO. 

Amis et al. [5] mentioned MPFL as a thin band of fascia extending from the area of MEF to the proximal part of the medial border of the patella. The appearance of the MPFL and its bulk varies from person to person and it may be very thin to identify. Mainly the researchers described the shape of the MPFL as triangular [20], but some named it ‘sail shaped’ [21] or hourglass-shaped [22]. We had not found any article which morphologically classified MPFL into three categories: triangular, rectangular, and two straps. We reported here 67.5% triangular, 20% rectangular, and 12.5% two-strap variant patterns of MPFL. 

## Conclusions

This study added knowledge about the exact origin and insertion of MPFL. The origin lies between the adductor tubercle and the medial epicondyle of the femur. For the insertion fibers of MPFL extended to the proximal medial half of the patella and the tendinous aponeurosis of VMO and VIM. The three variant patterns: triangular, rectangular, and two straps of MPFL are reported for the first time in this study. This knowledge will be helpful to the surgeons involved in the repair of the MPFL.

## References

[1] Stranding S (2016). Gray’s Anatomy, The Anatomical Basis of Clinical Practice, 41st edition.

[2] Conlan T, Garth WP Jr, Lemons JE (1993). Evaluation of the medial soft-tissue restraints of the extensor mechanism of the knee. J Bone Joint Surg Am.

[3] Feller JA, Feagin JA Jr, Garrett WE Jr (1993). The medial patellofemoral ligament revisited: an anatomical study. Knee Surg Sports Traumatol Arthrosc.

[4] Nomura E, Inoue M, Osada N (2005). Anatomical analysis of the medial patellofemoral ligament of the knee, especially the femoral attachment. Knee Surg Sports Traumatol Arthrosc.

[5] Amis AA, Firer P, Mountney J, Senavongse W, Thomas NP (2003). Anatomy and biomechanics of the medial patellofemoral ligament. Knee.

[6] Christiansen SE, Jacobsen BW, Lund B, Lind M (2008). Reconstruction of the medial patellofemoral ligament with gracilis tendon autograft in transverse patellar drill holes. Arthroscopy.

[7] Steensen RN, Dopirak RM, Maurus PB (2005). A simple technique for reconstruction of the medial patellofemoral ligament using a quadriceps tendon graft. Arthroscopy.

[8] Nomura E, Inoue M (2006). Hybrid medial patellofemoral ligament reconstruction using the semitendinous tendon for recurrent patellar dislocation: minimum 3 years' follow-up. Arthroscopy.

[9] Boden BP, Pearsall AW, Garrett WE Jr, Feagin JA Jr (1997). Patellofemoral instability: evaluation and management. J Am Acad Orthop Surg.

[10] Sallay PI, Poggi J, Speer KP, Garrett WE (1996). Acute dislocation of the patella. A correlative pathoanatomic study. Am J Sports Med.

[11] Pinskerova V, Maquet P, Freeman MA (2000). Writings on the knee between 1836 and 1917. J Bone Joint Surg Br.

[12] Kang HJ, Wang F, Chen BC, Su YL, Zhang ZC, Yan CB (2010). Functional bundles of the medial patellofemoral ligament. Knee Surg Sports Traumatol Arthrosc.

[13] Philippot R, Chouteau J, Wegrzyn J, Testa R, Fessy MH, Moyen B (2009). Medial patellofemoral ligament anatomy: implications for its surgical reconstruction. Knee Surg Sports Traumatol Arthrosc.

[14] Desio SM, Burks RT, Bachus KN (1998). Soft tissue restraints to lateral patellar translation in the human knee. Am J Sports Med.

[15] Hautamaa PV, Fithian DC, Kaufman KR, Daniel DM, Pohlmeyer AM (1998). Medial soft tissue restraints in lateral patellar instability and repair. Clin Orthop Relat Res.

[16] Smirk C, Morris H (2003). The anatomy and reconstruction of the medial patellofemoral ligament. Knee.

[17] Tuxøe JI, Teir M, Winge S, Nielsen PL (2002). The medial patellofemoral ligament: a dissection study. Knee Surg Sports Traumatol Arthrosc.

[18] Baldwin JL (2009). The anatomy of the medial patellofemoral ligament. Am J Sports Med.

[19] Mochizuki T, Nimura A, Tateishi T, Yamaguchi K, Muneta T, Akita K (2013). Anatomic study of the attachment of the medial patellofemoral ligament and its characteristic relationships to the vastus intermedius. Knee Surg Sports Traumatol Arthrosc.

[20] Viste A, Chatelet F, Desmarchelier R, Fessy MH (2014). Anatomical study of the medial patello-femoral ligament: landmarks for its surgical reconstruction. Surg Radiol Anat.

[21] Placella G, Tei MM, Sebastiani E (2014). Shape and size of the medial patellofemoral ligament for the best surgical reconstruction: a human cadaveric study. Knee Surg Sports Traumatol Arthrosc.

[22] Aframian A, Smith TO, Tennent TD, Cobb JP, Hing CB (2017). Origin and insertion of the medial patellofemoral ligament: a systematic review of anatomy. Knee Surg Sports Traumatol Arthrosc.

